# Physiological Response of *Tribolium castaneum* to CO_2_ Controlled Atmosphere Stress Under Trehalose Feeding

**DOI:** 10.3390/insects16080768

**Published:** 2025-07-26

**Authors:** Yuya Zhang, Shangrong Hu, Min Zhou, Xinyi Zhang, Liwen Guan, Yanfei Zhou, Jun Lv, Bin Tang

**Affiliations:** 1 College of Life and Environmental Sciences, Hangzhou Normal University, Hangzhou 311121, China; zyya1c@163.com (Y.Z.); 15850396510@163.com (S.H.); zhoumin810611@126.com (M.Z.); 2023210301033@stu.hznu.edu.cn (X.Z.); guanliwen1010@163.com (L.G.); 19858108489@163.com (Y.Z.); 2 School of Food Engineering, Moutai Institute, Renhuai 564507, China

**Keywords:** modified atmosphere grain storage, *Tribolium castaneum*, CO_2_ stress, trehalose, trehalase, cytochrome oxidase P450 enzyme

## Abstract

This study investigated the physiological regulatory mechanisms by which exogenous trehalose intake enhances the adaptation of the global stored-grain pest *T. castaneum* to high-concentration carbon dioxide (CO_2_) stress. By supplementing exogenous trehalose under high-CO_2_ controlled atmosphere stress, we measured the activities of key detoxification enzymes (e.g., carboxylesterase and cytochrome P450) and the levels of carbohydrate substances (e.g., glycogen, glucose, and trehalose). The results demonstrated that trehalose feeding significantly alleviated CO_2_ induced mortality in *T. castaneum* and prolonged their survival time. In terms of detoxification metabolism, a trehalose-rich diet significantly reduced the activities of cytochrome P450 and carboxylesterase, while the glucose content in the beetles decreased markedly. These findings indicate that trehalose accumulation mitigates physiological damage caused by high-CO_2_ stress in *T. castaneum*. Furthermore, exogenous trehalose intake did not disrupt carbohydrate metabolic homeostasis in the beetles, as trehalase activity and the levels of various carbohydrates remained relatively stable. This study elucidates the role of trehalose metabolism in *T. castaneum*’s adaptation to high-CO_2_ environments, providing a theoretical foundation for optimizing controlled atmosphere grain storage technology and developing novel pest control strategies.

## Simple Summary

As one of the most destructive stored-grain pests worldwide, the red flour beetle (*Tribolium castaneum*) causes severe damage to grain storage. In this study, we employed entomological research methods, including sublethal high-CO_2_-modified atmosphere stress treatment and enzyme kinetic assays, along with physiological, biochemical, and molecular biological approaches, to investigate the role of trehalose in *T. castaneum*’s response to elevated CO_2_ levels. By supplementing exogenous trehalose, we elucidated how trehalose metabolism aids this pest in adapting to high-CO_2_ environments. These findings provide novel insights for improving controlled atmosphere grain storage technology and developing more effective pest management strategies.

## 1. Introduction

Globally, postharvest grain losses caused by stored-product pests account for approximately 10% of total stored quantities annually, sometimes exceeding losses from field crop pests during cultivation [1]. Among these pests, *T. castaneum*, is one of the most notorious stored-grain pests worldwide. Its remarkable resilience and broad dietary adaptability enable *T. castaneum* to infest diverse storage environments, resulting in significant grain damage and substantial economic losses [2,3]. In the process of life, the adult insects will secrete a large number of harmful chemical pollutants, which will make the grain deteriorate and emit a pungent odor, and the quality of the grain will be reduced [4,5]. Additionally, *T. castaneum* produces metabolic byproducts such as feces and exuviae. These byproducts may trigger respiratory or dermatological diseases in humans [6]. Current pest control predominantly relies on chemical fumigants (e.g., phosphine and methyl bromide) and physical barrier technologies [7,8]. However, prolonged use of these chemicals has led to pest resistance, potentially linked to their diapause-related physiological adaptation mechanisms or specialized appendage structures [9,10,11,12]. Moreover, phosphine and methyl bromide contribute to hazardous pesticide residues and environmental pollution, posing risks to human health. Therefore, it is imperative to develop environmentally friendly and sustainable pest control strategies to address this issue.

Controlled atmosphere technology involves introducing nitrogen or carbon dioxide into sealed environments to reduce oxygen levels and create hypoxic conditions. This method is both energy-efficient and eco-friendly, making it a valuable pest control approach [9]. Low-oxygen environments effectively suppress aerobic respiration in stored-product pests, thereby impairing their growth, development, reproduction, and even causing mortality [13,14,15]. However, studies have shown that under short-term hypoxia, insects can adapt by regulating spiracle opening/closing and enhancing abdominal contractions to increase oxygen uptake. These adaptations allow them to cope with low-oxygen conditions. Concurrently, they reduce movement and slow developmental processes to reduce the risk of tissue damage and oxygen demand [16,17]. This suggests that hypoxia alone is insufficient because prolonged low-oxygen stress can induce adaptive resilience in stored-product pests [18,19]. As a result, these pests can survive for days or longer under hypoxic conditions [14,20]. Certain pests counteract hypoxia in various ways. They may elevate internal glucose and amino acid levels or boost metabolic activity to adapt to low-oxygen conditions [21]. Furthermore, extended exposure to high-CO_2_ concentrations may degrade grain protein content and quality [22], posing significant challenges for long-term storage sustainability. Therefore, it is crucial to understand pest adaptation mechanisms during controlled atmosphere treatments to effectively advance controlled atmosphere grain storage technologies.

Cytochrome P450, as a biological enzyme, plays crucial physiological roles in nearly all life stages of insects, participating not only in the synthesis pathways of molting hormones and juvenile hormones that regulate insect development but also in the degradation of toxic substances [23,24,25]. Similar to carboxylesterases (CarEs), its detoxification capability enables insects to counteract plant defensive compounds and insecticide toxicity, thereby conferring resistance [26,27].

Trehalose, a non-reducing disaccharide composed of two glucose units linked through an α,α-1,1-glycosidic bond, serves as the primary blood sugar in insects. It is hydrolyzed by trehalase into glucose, which serves as a direct energy source, while glycogen functions as a long-term energy reserve. These three compounds dynamically interconvert to maintain energy homeostasis and environmental adaptability in insects. Trehalose is ubiquitous in nature, with trehalose-metabolizing enzymes identified in various organisms, ranging from bacteria, yeast, and fungi to insects, higher plants, and even mammals [28,29]. Beyond its roles as an energy and carbon source, trehalose exhibits remarkable protective properties. Numerous studies have shown that trehalose not only functions as an energy and carbon source but also safeguards cell membranes and proteins from inactivation or denaturation caused by various stress conditions, such as desiccation, heat, and oxidative stress [30,31]. In insects, trehalose homeostasis is finely tuned in response to environmental challenges, with hemolymph trehalose levels being precisely adjusted to protect tissues and enhance stress adaptation [32,33,34]. These adaptive mechanisms have positioned trehalose metabolism as a key area of focus in stress physiology research. Notably, trehalose provides protection against xenobiotic toxicity in insects [35,36], and its metabolic pathways, particularly those involving trehalose-6-phosphate synthase (TPS) and trehalase (TRE), have emerged as promising targets for pest control strategies [37,38,39]. Furthermore, trehalose acts as a glucose source for chitin synthesis, with trehalase playing a crucial role as the initial enzyme in this pathway. Chitin, a vital component of the endophagophage, tracheal system, and body surface shell, is closely associated with insect growth, development, and even respiration [40]. Collectively, these findings emphasize trehalose’s dual role in energy metabolism and broader physiological regulation, underscoring its significance in insect biology. Additionally, studies on the administration of high concentrations of exogenous trehalose to *Acyrthosiphon pisum* have demonstrated its feasibility [41].

In addition, trehalose metabolism demonstrates significant regulatory interplay with endogenous hormonal signaling pathways. Insulin and adipokinetic hormone (AKH) serve as key metabolic regulators in insects, serving a similar function to the insulin–glucagon axis in regulating mammalian energy homeostasis [42,43]. Compelling evidence from *Drosophila melanogaster* models demonstrates this relationship: Targeted ablation of insulin-producing cells induces significant hypertrehalosemia, while genetic restoration of insulin production restores hemolymph trehalose levels to normal [44]. These findings establish insulin as a primary negative regulator of trehalogenesis. Given that AKH counteracts insulin signaling to mobilize energy reserves during stress, these two hormones likely synergistically regulate trehalose dynamics during hypoxic adaptation through reciprocal regulation of trehalose synthesis and catabolism. In this study, we investigated the physiological and molecular responses of the model insect *T. castaneum* to high-CO_2_ stress under exogenous trehalose supplementation. By administering trehalose-enriched diets, we systematically monitored key physiological parameters including survival rate, pupation rate, eclosion rate, carbohydrate profiles (glycogen, glucose, and trehalose), and trehalose-related enzyme activities. These comprehensive analyses aimed to elucidate the protective role of trehalose metabolism in enhancing CO_2_ tolerance. Furthermore, this work provides mechanistic insights into the adaptation strategies of stored-product pests to controlled atmosphere storage conditions, proposing a novel approach to overcome resistance development associated with current low-oxygen pest control technologies such as hypercapnic treatments.

## 2. Materials and Methods

### 2.1. Insect Source and Feeding Method

The *T. castaneum* used in this experiment were sourced from a laboratory-maintained colony at Hangzhou Normal University. Larvae were reared in an artificial climate chamber (temperature: 29 ± 1 °C, relative humidity: 65 ± 5% RH, photoperiod: 0 L:24 D) on a diet of whole wheat flour containing 5% yeast (Angel Yeast Co., Ltd., Yichang, China). Neutral Red, a vital cell dye widely used in biological experiments with minimal cellular damage and excellent biocompatibility, was employed in this study.

CO_2_ modified atmosphere method: Transfer the 8th instar larvae of *T. castaneum* with consistent growth to the culture chamber, add an appropriate amount of whole wheat flour containing 5% yeast as their diet, cover, and seal. Connect the catheter to the modified atmosphere mixer (QT-MIX-3, Changsha Changjin Technology Co., Ltd., Changsha, China) to introduce the set concentration of CO_2_ gas. Stop ventilation after the CO_2_ concentration reaches the preset level. Place the inlet and outlet catheters of the culture chamber within an artificial climate chamber for incubation and monitoring.

We selected 8th instar larvae for three different feeding treatments: (1) flour-only feeding; (2) 50% trehalose + 50% flour; (3) trehalose-only feeding. The three treatments were reared under normal atmospheric conditions and under an atmosphere of 95% CO_2_ and 5% air, respectively. About 50 larvae per feeding treatment were set up in one replicate, and 4 replicates were set for each treatment, with a total of 200 larvae in each treatment. The larvae were observed every 24 h until all larvae died, and the survival rate was counted and the average lifespan was calculated. After pupation and emergence, the pupation rate and emergence rate were counted. Criteria for determining mortality: After removal from the treatment environment for 5 h, the insects were stimulated with a brush, and those that did not respond were considered dead.

### 2.2. Main Experimental Instruments

The following instruments were utilized: Bioruptor Pico (Diagenode SA, Diagenode, Beiglum), microscope (Leica Microsystems Shanghai Trading Co., Ltd., Shanghai, China); Electronic balance (Mettler Toledo Instruments Shanghai Co., Ltd., Shanghai, China), ultra-low temperature centrifuge (Eppendorf, Hamburg, Germany); Spectrophotometer (Thermo Fisher Scientific, Waltham, MA, USA), water bath (Shanghai Jinghong Experimental Equipment Co., Ltd., Shanghai, China).

### 2.3. Cytochrome Oxidase P450 Enzyme Activity Detection

This experiment used the insect cytochrome P450 (CYP450) enzyme-linked immunosorbent assay (ELASA) kit (Shanghai Keaibo Biotechnology, Shanghai, China) for detection. Whole specimens of *T. castaneum* were crushed using a disruptor to obtain samples. Take approximately 0.03 g sample per replicate, with 4 replicates per treatment. After weighing, add 30 μL PBS and store in liquid nitrogen. Thaw to 2–8 °C before experiment. Add 270 μL PBS, homogenize, and centrifuge at 2500× *g* for 20 min to obtain supernatant. Set up standard and sample wells, adding 50 μL of standards of different concentrations to standard wells. Include blank wells (without sample and enzyme label), and sample wells to be tested. Add 40 μL of diluent and 10 μL of the sample to be tested (5-fold dilution) to the sample well. Add 100 μL enzyme label to all wells except blanks, seal, and incubate at 37 °C for 60 min. Dilute 20× wash solution with distilled water. Remove seal, discard liquid, air-dry, wash each well 5 times with wash solution, and pat dry. Add 50 μL of chromogen A and B to each well, mix, and incubate at 37 °C for 15 min. Add 50 μL stop solution to each well (color changes from blue to yellow). Measure absorbance at 450 nm with blank well zeroing. Complete assay within 15 min after adding stop solution.

### 2.4. Carboxyesterase Detection

Prepare four biological replicates for each treatment group, with each replicate containing approximately 0.03 g of sample. Add extraction buffer (Reagent I) to each sample tube at a 1:10 (*w*/*v*) ratio of sample mass (g) to buffer volume (mL); then homogenize the mixture using a mechanical homogenizer maintained in an ice bath. Centrifuge the homogenate at 12,000× *g* for 30 min at 4 °C. Carefully transfer the supernatant to sterile polypropylene centrifuge tubes using a calibrated pipette, avoiding cellular debris carryover. Quantify carboxylesterase (CarE) activity using a commercial colorimetric assay kit (Nanjing Jiancheng Bioengineering Institute, Nanjing, China) according to the manufacturer’s protocol. Calculate enzymatic activity using the standardized formula provided with the kit, ensuring absorbance measurements are normalized to total protein concentration.

### 2.5. Determination of Sugar Content Such as Glycogen, Glucose, Trehalose, etc.

Each replicate consists of fifteen samples of *T. castaneum*, and a total of four replicates are included for each treatment group. After homogenizing the samples, add 1 mL of PBS (pH 7.0), and sonicate for 30 min. Centrifuge at 4 °C for 20 min at a speed of 1000× *g*, and collect the supernatant for the detection of trehalose and glycogen content. Take another 350 μL of the supernatant, centrifuge at 4 °C for 60 min at a speed of 20,800× *g*, and collect both the supernatant and the pellet for measuring glucose content.

The anthrone method was used to detect the content of trehalose. Mix 30 μL of 1% H_2_SO_4_ with 30 μL of the sample and incubated in a 90 °C water bath for 10 min. Then, cool the mixture in an ice bath for 3 min, add 30 μL of 30% KOH, and the water bath at 90 °C was heated again, with ice bath for 3 min. Then, add 600 μL of developer (a mixture of 0.02 g anthrone and 100 mL of 80% H_2_SO_4_) to the mixture; after 10 min in a 90 °C water bath, it was placed in an ice bath for cooling. Finally, measure the absorbance at a wavelength of 630 nm. The detection methods for glycogen and glucose are basically the same, but trehalose requires taking 160 μL of sample first, adding 32 μL of 0.1 U/L starch glucosidase, and taking a 40 °C water bath for 4 h. The glucose (Go) detection kit (Lot No. SLCD8160, Sigma, St. Louis, MO, USA) is used to determine the substance content in the mixed solution and glucose sample. After the reaction is complete, add 12 N H_2_SO_4_ to terminate the reaction and measure its absorbance value at a wavelength of 540 nm.

### 2.6. Determination of Trehalase Activity

For the determination of trehalase activity, homogenization was carried out using stainless steel balls, subsequently followed by the addition of 1 mL PBS (pH 7.0) and ultrasonic disruption for 30 min. The mixture was subjected to centrifuged at 1000× *g* for 20 min at 4 °C. A 350 μL aliquot of the supernatant was collected and subjected to ultracentrifugation at 20,800× *g* for 60 min at the same temperature. The resulting supernatant was used to measure soluble trehalase activity and protein concentration. Simultaneously, the pellet was resuspended in 300 μL PBS for the determination of membrane-bound trehalase activity and protein concentration.

Next, 60 μL of the sample was mixed with 75 μL of 40 mM trehalose and 165 μL PBS; then incubated at 37 °C for 60 min. The reaction was terminated by heat inactivation at 100 °C for 5 min. A 130 μL aliquot of the mixture was analyzed using a Glucose (GO) Assay Kit (Lot No. SLCD8160, Sigma, St. Louis, MO, USA) to quantify trehalase activity. After the reaction, 260 μL of 12 N H_2_SO_4_ was added to stop the reaction, and absorbance was measured at 540 nm. Finally, protein content was determined using a BCA Protein Assay Kit (Cat No. P0006, Beyotime, Shanghai, China).

### 2.7. Data Analysis and Plotting

Data were organized using Microsoft Excel, figures were generated with GraphPad Prism 9.0.0, and statistical analyses were performed using IBM SPSS Statistics 23.0. Differences between treatment and control groups were evaluated by Student’s *t*-test. A significant difference was denoted by * when *p* < 0.05, a highly significant difference by ** when *p* < 0.01, and no significant difference (ns) when *p* > 0.05.

## 3. Results

### 3.1. Preference of T. castaneum for Trehalose Intake

**Figure 1:**
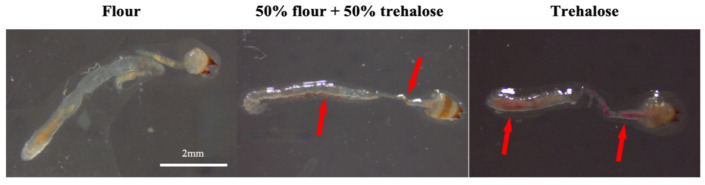
Anatomical diagram of the *T. castaneum* digestive tract after feeding diets with different trehalose ratios.

Trehalose was mixed with Neutral Red at a defined ratio, dissolved, dried, and ground into a powder for feeding *T. castaneum*. The digestive tracts of the beetles were dissected to observe whether selective ingestion of trehalose occurred. The dissection results are shown in Figure 1. After feeding on flour, 50% flour + 50% trehalose (stained with Neutral Red), or trehalose (stained with Neutral Red), red-stained trehalose accumulated in the midgut and hindgut of the latter two treatment groups (indicated by red arrows in Figure 1) under normoxic conditions. This indicates that *T. castaneum* can ingest trehalose without exhibiting preferential feeding behavior.

### 3.2. Effects of Feeding Trehalose on Pupation and Eclosion of T. castaneum

**Figure 2:**
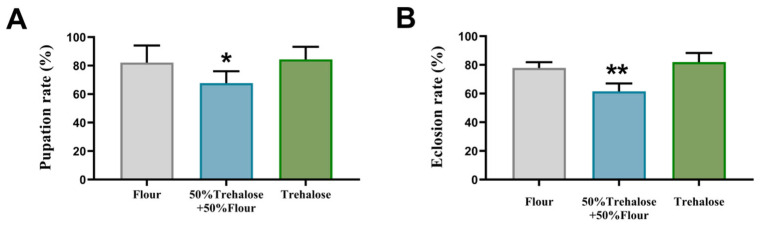
Pupation rate and emergence rate of *T. castaneum* after feeding different trehalose ratio diets. (**A**) Pupation rate, (**B**) Eclosion rate. Value are presented as the means ± SE. *: *p* < 0.05, **: *p* < 0.01 (independent samples *t*-test).

Under normoxic rearing conditions, *T. castaneum* larvae were fed three distinct dietary regimens to assess developmental impacts. As shown in Figure 2, the mixed flour–trehalose diet (50% flour + 50% trehalose) significantly reduced pupation rates by 14.4% compared to the flour-only control (Figure 2A). In contrast, larvae fed exclusively trehalose showed no significant difference in pupation rates relative to controls. A parallel trend was observed in eclosion success: The mixed diet group exhibited a 20.0% reduction in eclosion rate, while the full trehalose diet group remained comparable to controls (Figure 2B).

### 3.3. The Effect of a Trehalose Diet on T. castaneum Under Controlled Atmosphere Treatment

**Figure 3:**
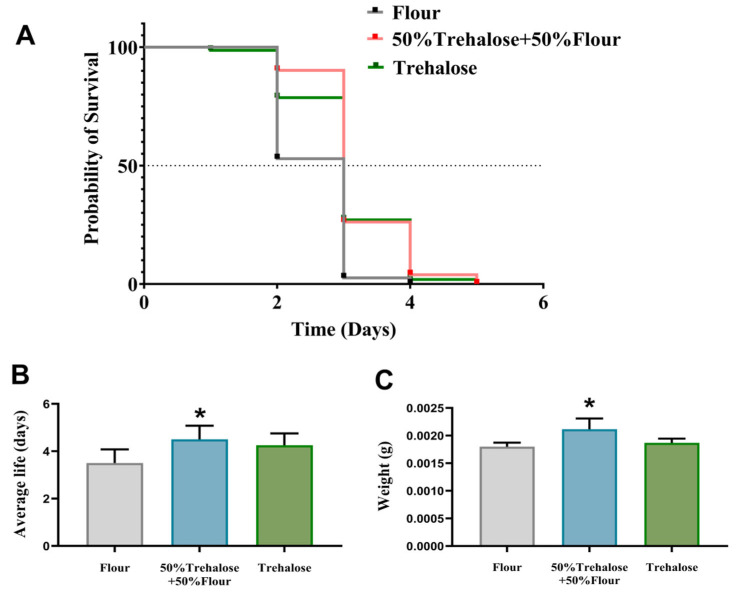
Line plots showing the death, mean life span, and mean body weight of *T. castaneum* after 48 h of CO_2_ controlled atmosphere combined with different dietary treatments. (**A**) Probability of Survival, (**B**) Average life, (**C**) Weight. Value are presented as the means ± SE. *: *p* < 0.05 (independent samples *t*-test).

We conducted a 95% CO_2_ atmosphere treatment on three different dietary regimens of *T. castaneum* and observed their mortality rate and weight changes. The mortality results are shown in Figure 3. Compared with the group fed on flour alone, the treatment group with a diet containing trehalose had a longer survival time (Figure 3A). Supplementation with 50% trehalose in the diet significantly extended the mean lifespan of *T. castaneum* by 28% versus the flour-only control (Figure 3B), concurrent with a 17% elevation in body weight gain (Figure 3C).

### 3.4. Effects of Trehalose Diet Under Controlled Atmosphere on Detoxification Enzyme Activity of T. castaneum

**Figure 4:**
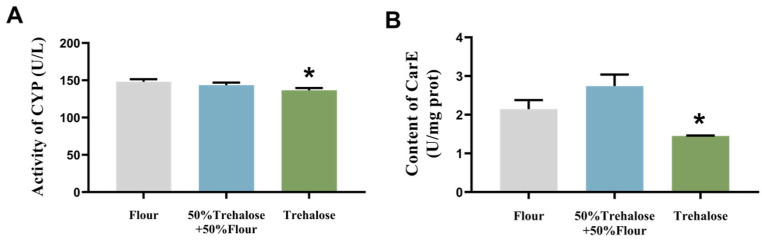
The activity of *T. castaneum* detoxification enzymes after 48 h of treatment with CO_2_ controlled atmosphere combined with different diets. (**A**) Activity of CYP, (**B**) Content of CarE. Value are presented as the means ± SE. *: *p* < 0.05 (independent samples *t*-test).

Under 95% CO_2_ atmosphere treatment, both trehalose-supplemented groups exhibited reduced cytochrome P450 activity compared to the flour-diet control. The full-trehalose diet group showed a significant 7% decrease (Figure 4A). For carboxylesterase activity, the mixed diet group (50% flour + 50% trehalose) increased relative to the control, whereas the full-trehalose group demonstrated a pronounced 32% reduction (Figure 4B).

### 3.5. The Effect of Trehalose Diet on Trehalose Metabolism in T. castaneum Under Controlled Atmosphere Treatment

**Figure 5:**
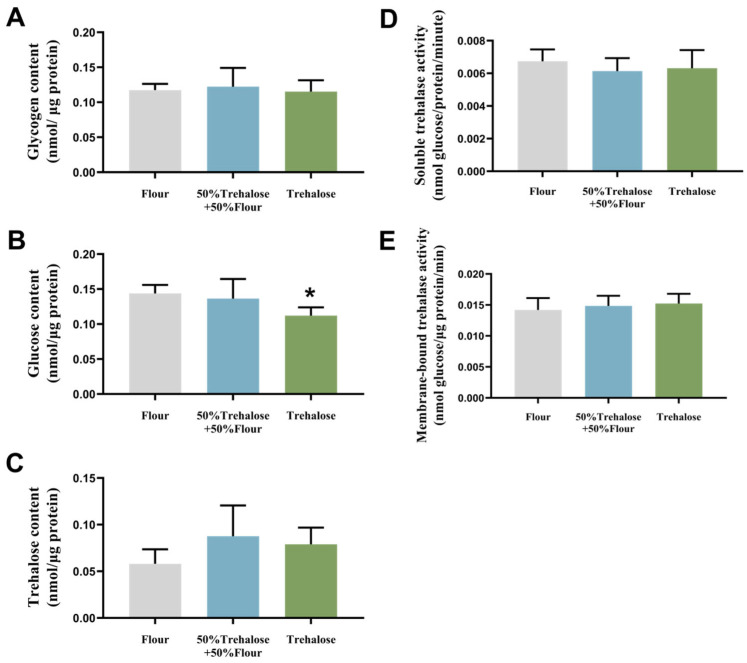
Carbohydrate content and trehalase activity of *T. castaneum* after 48 h of CO_2_ controlled atmosphere combined with different dietary treatments. (**A**) Glycogen content, (**B**) Glucose content, (**C**) Trehalose content, (**D**) Soluble trehalose activity, (**E**) Membrane-bound trehalose activity. Value are presented as the means ± SE. *: *p* < 0.05 (independent samples *t*-test).

Under a 95% CO_2_ controlled atmosphere stress condition, we measured the sugar content after 48 h of trehalose feeding to ensure complete absorption of the food and to eliminate the interference from intestinal residual unabsorbed sugar. As shown in Figure 5, there was little variation in glycogen content among the three dietary groups (Figure 5A). When subjected to trehalose dietary interventions, glucose levels decreased across all treatment groups. Specifically, the full-trehalose diet induced a 21% decrease in glucose levels compared to the flour-fed controls, while the mixed diet (50% flour + 50% trehalose) also exhibited reduced glucose content (Figure 5B). Regarding trehalose content, both treatment groups fed with trehalose-containing diets had higher trehalose levels than the control group fed with the flour diet, although the differences were not statistically significant (Figure 5C). The changes in trehalose enzyme activity deviated from the changes observed in trehalose content. The soluble trehalose enzyme activity of the two treatment groups decreased compared to the control group fed on flour (Figure 5D), whereas the membrane-bound trehalose enzyme activity increased (Figure 5E); however, these changes were also not statistically significant.

## 4. Discussion

Trehalose, which is the predominant sugar in the hemolymph of insects, serves not only as an energy source and is involved in fundamental physiological processes such as energy metabolism, chitin biosynthesis, and stress adaptation [45] but also mediates adaptive responses to environmental stressors like drought, hypoxia, and xenobiotic toxicity through its unique chaperone-like function [46,47,48,49,50]. As early as 2003, Chen et al. [51] demonstrated that trehalose synthesis in HEK-293 cells significantly suppresses hypoxia-induced protein aggregation, thereby improving the cells’ tolerance to hypoxia. This finding suggests that trehalose metabolism may play a pivotal role in pest adaptation to controlled atmosphere stress environments, such as high-CO_2_ conditions. By elucidating the molecular mechanisms underlying high-CO_2_ resistance in stored-grain pests like *T. castaneum*, this study aims to overcome resistance bottlenecks associated with traditional low-oxygen pest control methods, thus providing a theoretical foundation for developing novel pest management strategies aimed at regulating trehalose homeostasis.

The content of trehalose is intricately linked to the growth and developmental processes of both animals and plants. OHara L. E. et al. [52] postulate that trehalose-6-phosphate (T6P), acting as a precursor in the biosynthetic pathway, plays a crucial role in modulating plant growth and development in accordance with carbon availability. By inhibiting SnRK1, T6P facilitates biosynthetic reactions, thereby holding significant implications for crop yield and productivity. For instance, OHara et al. [52] have observed its influence in wheat grain development and potato tuber growth. Li et al. [53] found that appropriate intake of trehalose can have a positive impact on the development and reproduction of ladybugs. Dogan and Tellis indicated that the content of trehalose is associated with insect development [54,55]. Jia et al. [56] caused a decrease in trehalose content in M. separator by inhibiting the TPS gene, resulting in insect deformities and increased mortality. However, in this study, under conventional oxygen conditions, there were no notable changes in the pupation and eclosion rates of *T. castaneum* after feeding with trehalose. This indicates that the trehalose diet does not have a significant impact on the abnormal development process of *T. castaneum* under conventional cultivation conditions. Nonetheless, it is worth noting that, in the mixed diet treatment group, the abnormal development of *T. castaneum* was affected to some extent, which may be related to unclear changes in the digestion of the mixed diet. In conclusion, the impact of exogenous trehalose feeding on the growth and development of *T. castaneum* is limited, potentially due to its inherent regulation of trehalose metabolism.

In this study, we explored the regulatory effect of exogenous trehalose on *T. castaneum* under 95% CO_2_ stress. High-CO_2_ levels cause respiratory retardation, oxidative stress, and energy metabolism disorders in insects [57]. We found that trehalose supplementation significantly improved the beetle’s living conditions and extended its lifespan compared to the control group. This aligns with findings in *Caenorhabditis elegans*, suggesting a cross-species mechanism of action for trehalose in maintaining energy homeostasis [58,59]. Analysis revealed multiple pathways for trehalose’s protective effect. It may regulate key transcription factors like DAF-16 via the insulin/IGF-1 signaling pathway (IIS), enhancing stress resistance [60]. Additionally, the mediation of ILP2 signaling results in the inhibition of trehalose synthesis and enhancement of its decomposition, leading to the rapid consumption of both trehalose and its decomposition product, glucose, within cells, thereby significantly decreasing their steady-state levels [57]. As early as 2003, scientists discovered that HEK-293 cells capable of synthesizing trehalose exhibited enhanced adaptation to hypoxia by mitigating protein aggregation, thereby validating the unique protective role of trehalose under hypoxic stress [61]. Notably, this study also revealed that trehalose feeding significantly reduced the activities of detoxifying enzymes (CYP450 and carboxylesterase) in *T. castaneum*. This phenomenon suggests that trehalose may diminish the metabolic demand for detoxification systems by either reducing oxidative damage or optimizing energy allocation. These findings provide a novel perspective for understanding the stress-resistance mechanisms of trehalose and are also consistent with the results reported by Jingjing et al. [62] and Hiroko et al. [63] regarding trehalose’s role in enhancing insect starvation resistance.

Trehalose-6-phosphate synthase (TPS) and trehalose synthase (TRE) are pivotal in regulating trehalose metabolism in insects, with indirect effects on chitin metabolism, as highlighted by Wang et al. [64]. Yoshida et al. [65] discovered that fruit flies with TRE deficiency perish due to excessive trehalose accumulation, whereas TPS deficiency mitigates this effect. Tetsuo et al. [66] demonstrated that excessive trehalose accumulation impairs the adaptation and feeding capabilities of insects in harsh environments. Yang et al. [67] proposed that trehalose can be catalyzed into glucose, which is subsequently absorbed and utilized by insect tissue cells. Collectively, these findings underscore the insects’ relatively flexible mechanism for regulating trehalose metabolism, as pointed out by Tellis et al. [55]. Notably, our study under 95% CO_2_ controlled atmosphere conditions revealed that a mixed diet of 50% flour and 50% trehalose outperformed a full trehalose diet in terms of average lifespan and body weight. This indicates that a diet exclusively composed of trehalose may not be wholly beneficial for *T. castaneum* larvae, as excessive trehalose accumulation and starch deficiency could adversely affect their survival and development. The potential reasons for this phenomenon may include: (1) The high-CO_2_ environment may induce alterations in the insect’s basal metabolism, necessitating a more balanced supply of carbon sources to sustain life activities. (2) Starch substances may possess unique energy supply advantages under hypoxic conditions, contributing to the enhanced survival and growth of the insects. (3) Maintaining moderate levels of trehalose can not only provide stress protection but also prevent metabolic imbalances, thereby optimizing the overall physiological state of the insects.

Wang et al. fed *A. pisum* with trehalose and observed an increase in glucose content in their diet high in trehalose [41]. However, in the study, a significant decrease in glucose content was noted in *T. castaneum* fed solely on trehalose. Therefore, we speculate that the effects of exogenous trehalose intake may differ among insect species. This variation could be attributed to the direct transport and utilization of trehalose, by passing the process of decomposition and resynthesis. The results of trehalose enzyme activity assays revealed no significant changes in the activity of either soluble or membrane-bound trehalose enzymes. This further supports the hypothesis that trehalose may have been transported directly, rather than decomposed and resynthesized, after its acquisition. Overall, under stress conditions induced by a 95% CO_2_-modified atmosphere, the metabolism of trehalose in *T. castaneum* did not exhibit significant changes. The activity of trehalose enzymes remained stable, and sugar content also remained relatively constant. These observations may be related to the metabolic regulatory mechanisms of *T. castaneum* itself; however, further verification of the expression of its metabolic pathways is required.

## 5. Conclusions

In this paper, we conducted a systematic metabolic analysis to investigate the effects of trehalose as an exogenous nutritional intervention on the storage pest *T. castaneum* under high-CO_2_-modified atmosphere conditions. Our findings revealed the dual effects of trehalose on this pest in high-CO_2_ environments: it promotes survival while inhibiting detoxification. This discovery offers a novel perspective on metabolic intervention for pest control. Looking ahead, through the implementation of a collaborative strategy that integrates “metabolic target modified atmosphere technology,” we have the potential to develop more efficient and low-toxicity green grain storage solutions, thereby contributing to global food security.

## Data Availability

The original contributions presented in this study are included in the article. Further inquiries can be directed to the corresponding author.

## Abbreviations

The following abbreviations are used in this manuscript:

TPS	Trehalose-6-phosphate synthase
TRE	Trehalase
AKH	Adipokinetic hormone
PBS	Phosphate buffered saline
CYP450	Cytochrome P450
CarE	Carboxylesterase
T6P	Trehalose-6-phosphate
IIS	Insulin/IGF-1 signaling pathway
